# Pneumococcal Bacteremia Requiring Hospitalization in Rural Thailand: An Update on Incidence, Clinical Characteristics, Serotype Distribution, and Antimicrobial Susceptibility, 2005–2010

**DOI:** 10.1371/journal.pone.0066038

**Published:** 2013-06-26

**Authors:** Julia Rhodes, Surang Dejsirilert, Susan A. Maloney, Possawat Jorakate, Anek Kaewpan, Prasert Salika, Thantapat Akarachotpong, Prabda Prapasiri, Sathapana Naorat, Peera Areerat, Asadang Ruayajin, Pathom Sawanpanyalert, Pasakorn Akarasewi, Leonard F. Peruski, Henry C. Baggett

**Affiliations:** 1 International Emerging Infections Program, Global Disease Detection Regional Center, Thailand Ministry of Public Health – United States Centers for Disease Control and Prevention Collaboration, Nonthaburi, Thailand; 2 National Institute of Health, Ministry of Public Health, Nonthaburi, Thailand; 3 Sa Kaeo Provincial Health Office, Sa Kaeo, Thailand; 4 Nakhon Phanom Provincial Health Office, Nakhon Phanom, Thailand; 5 Bureau of Epidemiology, Ministry of Public Health, Nonthaburi, Thailand; Robert Koch Institut, Germany

## Abstract

**Background:**

*Streptococcus pneumoniae* is an important cause of morbidity and mortality in Southeast Asia, but regional data is limited. Updated burden estimates are critical as pneumococcal conjugate vaccine (PCV) is highly effective, but not yet included in the Expanded Program on Immunization of Thailand or neighboring countries.

**Methods:**

We implemented automated blood culture systems in two rural Thailand provinces as part of population-based surveillance for bacteremia. Blood cultures were collected from hospitalized patients as clinically indicated.

**Results:**

From May 2005– March 2010, 196 cases of pneumococcal bacteremia were confirmed in hospitalized patients. Of these, 57% had clinical pneumonia, 20% required mechanical ventilation, and 23% (n = 46) died. Antibiotic use before blood culture was confirmed in 25% of those with blood culture. Annual incidence of hospitalized pneumococcal bacteremia was 3.6 per 100,000 person-years; rates were higher among children aged <5 years at 11.7 and adults ≥65 years at 14.2, and highest among infants <1 year at 33.8. The median monthly case count was higher during December–March compared to the rest of the year 6.0 vs. 1.0 (p<0.001). The most common serotypes were 23F (16%) and 14 (14%); 61% (74% in patients <5 years) were serotypes in the 10-valent PCV (PCV 10) and 82% (92% in <5 years) in PCV 13. All isolates were sensitive to penicillin, but non-susceptibility was high for co-trimoxazole (57%), erythromycin (30%), and clindamycin (20%).

**Conclusions:**

We demonstrated a high pneumococcal bacteremia burden, yet underestimated incidence because we captured only hospitalized cases, and because pre-culture antibiotics were frequently used. Our findings together with prior research indicate that PCV would likely have high serotype coverage in Thailand. These findings will complement ongoing cost effectiveness analyses and support vaccine policy evaluation in Thailand and the region.

## Introduction

In 2009, The Hib and Pneumococcal Global Burden of Disease Study Team estimated that *Streptococcus pneumoniae* caused nearly 5.5 million meningitis, sepsis, and pneumonia cases and >185,000 deaths in Southeast Asia annually, but noted that regional prevention decisions, ‘will need to be made on the basis of limited regional data’ [1]. Similarly, The Asian Strategic Alliance for Pneumococcal Disease Prevention concluded that ‘pneumococcal disease is an important cause of morbidity and mortality in the Asian region’ and highlighted the ‘urgent’ need for ‘more substantial studies’ describing invasive pneumococcal disease burden in the Asia region [2].

Although WHO recommends pneumococcal conjugate vaccine (PCV) vaccination even in the absence of local data, policymakers often require local data to weigh costs and benefits [3]. Besides a paucity of local data, policymakers in Southeast Asian countries are faced with weighing the potential benefits of PCV against those of several other effective vaccines, including those against rotavirus, influenza, and human papillomavirus. Currently, PCV is not included in the National Expanded Programs of Immunization of Thailand or neighboring countries, though PCV it is available on the private market in Thailand [4].

Cost reductions are anticipated as the PCV Advanced Market Commitment is expected to increase demand, mass production, and manufacturer competition [5]. We aim to provide local and regional data to inform decision making as these changes occur. Previously, we published the first population-based estimates of pneumococcal bacteremia incidence in Southeast Asia [6]. The purpose of this report is to update these estimates and to contribute to an evidence base upon which sound policy decisions can be made.

## Methods

### Setting

The Thailand International Emerging Infections Program (IEIP) is part of a collaboration between the Thailand Ministry of Public Health and the U.S. Centers for Disease Control and Prevention. We conduct surveillance for community-acquired pneumonia requiring hospitalization in Sa Kaeo and Nakhon Phanom provinces, where the combined populations total 1.2 million, including >80,000 children <5 years [7]. Pneumonia surveillance is conducted at all 18 district and military hospitals and both provincial hospitals. Bloodstream infection surveillance began in all hospitals in May 2005 in Sa Kaeo and in November 2005 in Nakhon Phanom with the implementation of automated blood culture systems. Published detailed descriptions of these surveillance systems are available, [6], [8].

### Patients

Physicians request blood cultures from hospitalized patients as clinically indicated. Limited data (age, province, and pre-blood culture antibiotic use) are available for patients who are in the bloodstream infection surveillance system only. Detailed clinical and demographic information is available for patients who were also captured in the IEIP pneumonia surveillance system.

### Specimen Collection and Laboratory Methods

Blood cultures collected at district hospitals were transported at 15–30°C within 24 hours and processed at provincial hospital laboratories using the BactT/ALERT® 3D microbial detection system (bioMeriéux). Each blood specimen was divided between a bottle optimized for standard aerobic growth and a bottle for enhanced growth of fastidious pathogens, with priority given to inoculating at least 10 ml from adults and 4 ml from children <5 years of age into the standard bottle. Bottles that signaled positive growth (alarm-positive) were subcultured using standard methods [9].

Serotyping was performed using multiplex polymerase chain reaction (PCR) [10]. For isolates that could not be typed by this method, Quellung serotyping was done at the Streptococcus Reference Laboratory, U.S. Centers for Disease Control and Prevention in Atlanta, Georgia. Antimicrobial susceptibilities were determined by the disk-diffusion method with MIC values of penicillin and cefotaxime determined by Etest (AB Biodisk). Penicillin susceptibility interpretations used the 2008 Clinical and Laboratory Standards Institute guidelines for non-meningitis isolates: susceptible, ≤2 µg/ml; intermediate, 4 µg/ml; resistant ≥8 µg/ml [11].

Antibiotic use before blood culture was determined by a serum disc assay. A filter paper disc coated with patient serum was placed onto a Mueller-Hinton agar plate inoculated with pan-sensitive *Staphylococcus aureus* (ATCC 9144) and growth inhibition was measured after 24 hours incubation at 35–37°C, [6], [12].

### Statistical Analysis

We calculated the observed (i.e., minimum) incidence of pneumococcal bacteremia requiring hospitalization using person-years of follow-up based on province specific annual population estimates from the National Economic and Social Development Board of Thailand [7]. Population estimates for infants were obtained by applying the proportion of children <1 year old among all children <5 years old nationally to the surveillance population aged <5 years [13]. Exact 95% confidence intervals (CI) were calculated based on a Poisson distribution. Statistical analyses were done using SAS version 9.2 (SAS Institute Inc., Cary, NC, USA).

## Results

From May 2005 through March 2010, 5,118 of 67,516 (7.6%) blood cultures performed in these 2 provinces were positive for any pathogen, *S. pneumoniae* was isolated from the blood of 196 patients: 92 from Sa Kaeo province and 104 from Nakhon Phanom. *S. pneumoniae* was isolated from 0.33% of 27,655 blood cultures in Sa Kaeo compared to 0.26% (104/39,855) in Nakhon Phanom.

Among all patients with blood cultures, 25% were less than 5 years old and 25% were 65 years and older (Table 1). Deaths were more common among patients with pneumococcal bacteremia from Sa Kaeo: 37% (n = 34) in SK vs. 12% (n = 12) in NP. Additional clinical details were available for 130 of 196 patients with pneumococcal bacteremia who were also captured by IEIP’s pneumonia surveillance system. Of these, 86% had respiratory symptoms, 100% had evidence of acute infection and 86% (111/130) had both, and thus met IEIP’s criteria for clinical pneumonia. Pneumonia (ICD-10 codes J14–J19) was the discharge diagnosis for 45% (58/130) and septicemia (ICD-10 code A41.9) for another 22% (29/130). Case-patients from Sa Kaeo were more likely to receive oxygen or be intubated: oxygen use (77% in SK vs. 50% in NP, p = 0.001), intubation (46% in SK vs. 16% in NP, p<0.001), which is consistent with the higher case fatality rate observed in Sa Kaeo.

**Table 1:** Clinical characteristics of patients with blood culture and hospitalized pneumococcal bacteremia cases in rural Thailand, May 2005–March 2010.

	All patients with blood culture N = 67,516		All pneumococcal bacteremia cases N = 196		Pneumococcal bacteremia cases captured in pneumonia surveillance N = 130	
	N	%	N	%	N	%
**Age**						
<5	16,908	25.0	40	20.4	26	19.9
5–19	6,532	9.7	20	10.2	12	9.2
20–49	14,700	21.8	50	25.5	31	23.7
50–64	12,341	18.3	36	18.4	26	19.9
65+	17,029	25.2	50	25.5	36	27.5
**Any respiratory symptoms**						
Cough	–	–	–	–	94	71.8
Dyspnea	–	–	–	–	70	53.4
Tachynea	–	–	–	–	29	22.8
**Evidence of acute infection**						
Documented Fever	–	–	–	–	77	58.8
Fever History	–	–	–	–	115	87.8
Elevated White Blood Cell Count	–	–	–	–	72	55.0
**Evidence of complicated illness**						
Oxygen	–	–	–	–	83	63.4
Intubation	–	–	–	–	40	30.5
**Outcome**						
Discharge	–	–	110	56.1	81	61.8
Transfer	–	–	26	13.3	12	9.2
Death	–	–	46	23.5	31	23.7
Self-discharge	–	–	9	4.6	7	5.3
Missing	–	–	5	2.5	0	0.0

Antibiotic use before blood culture was common; 25.4% (11,123/43,720) of those tested had serum antimicrobial activity. Among pneumococcal bacteremia cases, serum antimicrobial activity was found in only 5 of the 135 tested (3.7%).

**Figure 1:**
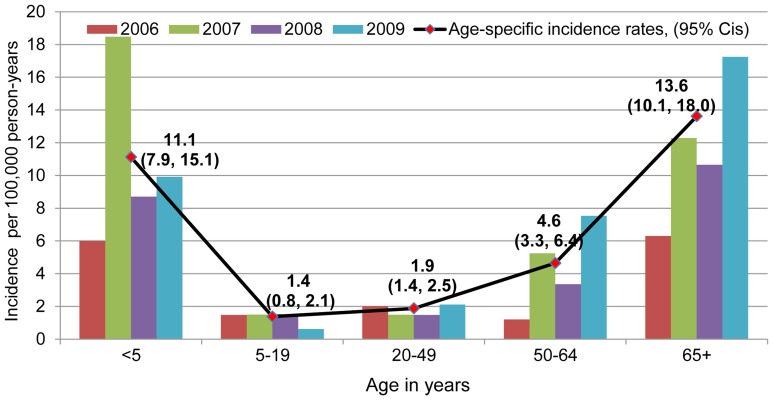
Hospitalized pneumococcal bacteremia incidence rates by year and age in rural Thailand, May 2005 to March 2010. Overall incidence 3.5 per 100,000 person-years, 95% CI (3.1, 4.1).

Hospitalized pneumococcal bacteremia incidence rates were highest among young children and older adults and varied by year (Figure 1). Overall incidence ranged from 2.3 per 100,000 person-years in 2006 to 4.1 in 2009 (data for 2005 and 2010 were incomplete and not considered). Among children less than 5 years old, the highest annual incidence rate was observed in 2007: 18.5 per 100,000. Among infants <1 year old, the average annual incidence was 33.8 per 100,000 (95% CI 21.4, 50.7).

**Figure 2:**
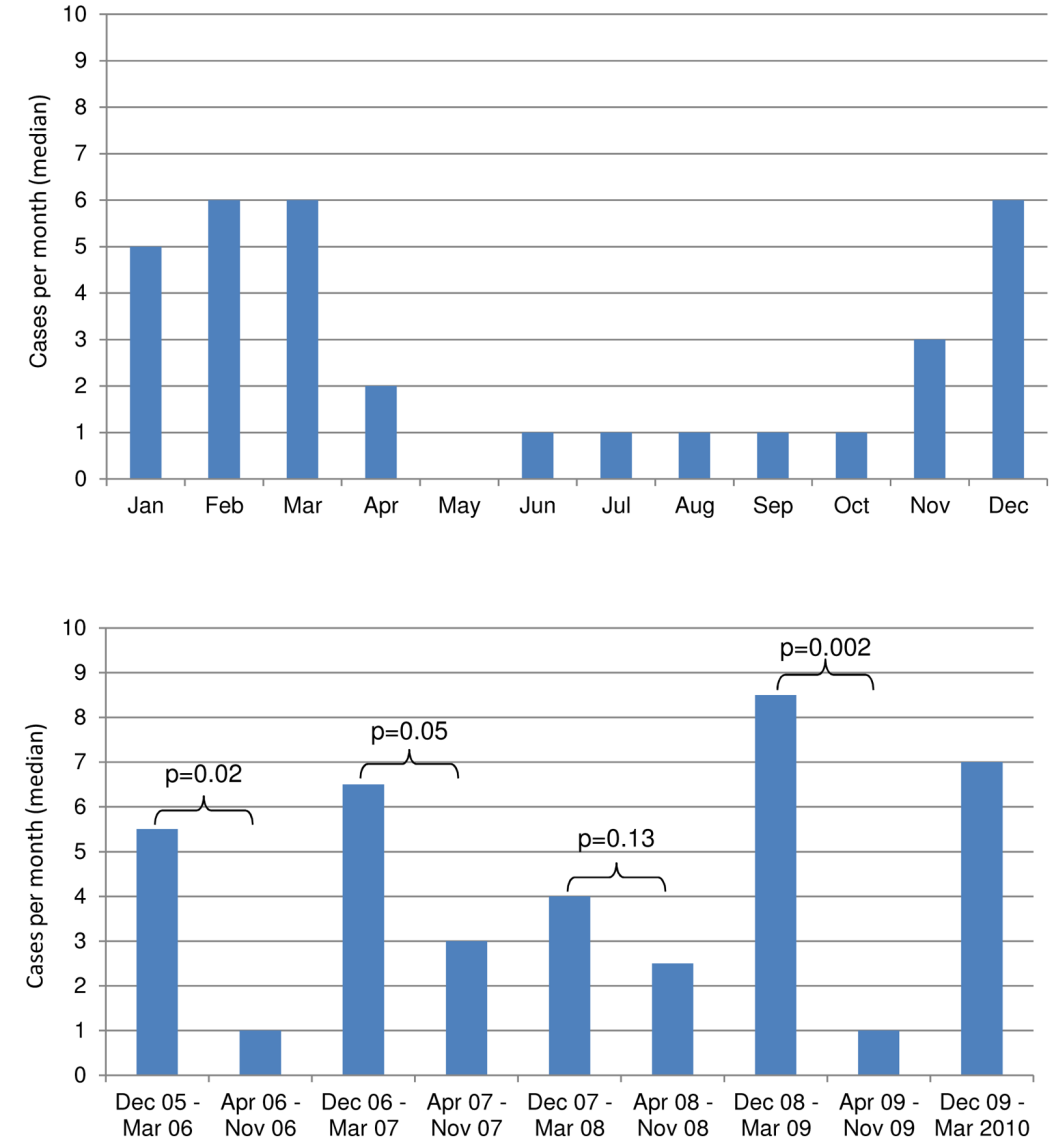
Hospitalized pneumococcal bacteremia in 2 rural Thai provinces, May 2005-Mar 2010: a. Median monthly case counts, b. Median monthly case counts during four consecutive December-March vs. April-November periods^*^. *p-values represent Wilcoxon Rank Sum Test comparisons.

The median number of pneumococcal bacteremia cases per month was significantly higher during December through March compared to the rest of the year: 6.0 cases per month during December-March vs. 1.0 during April-November (p<0.001) (Figure 2). This difference was observed in both Sa Kaeo and Nakhon Phanom provinces (data not shown).

Serotyping was completed for 191 (97%) of 196 isolates and 39 (98%) of 40 isolates from children <5 years old. Serotypes 14 and 23F were most common among both children and adults; by contrast, serotype 3 was common among adults, but not found in children <5 years.

**Figure 3:**
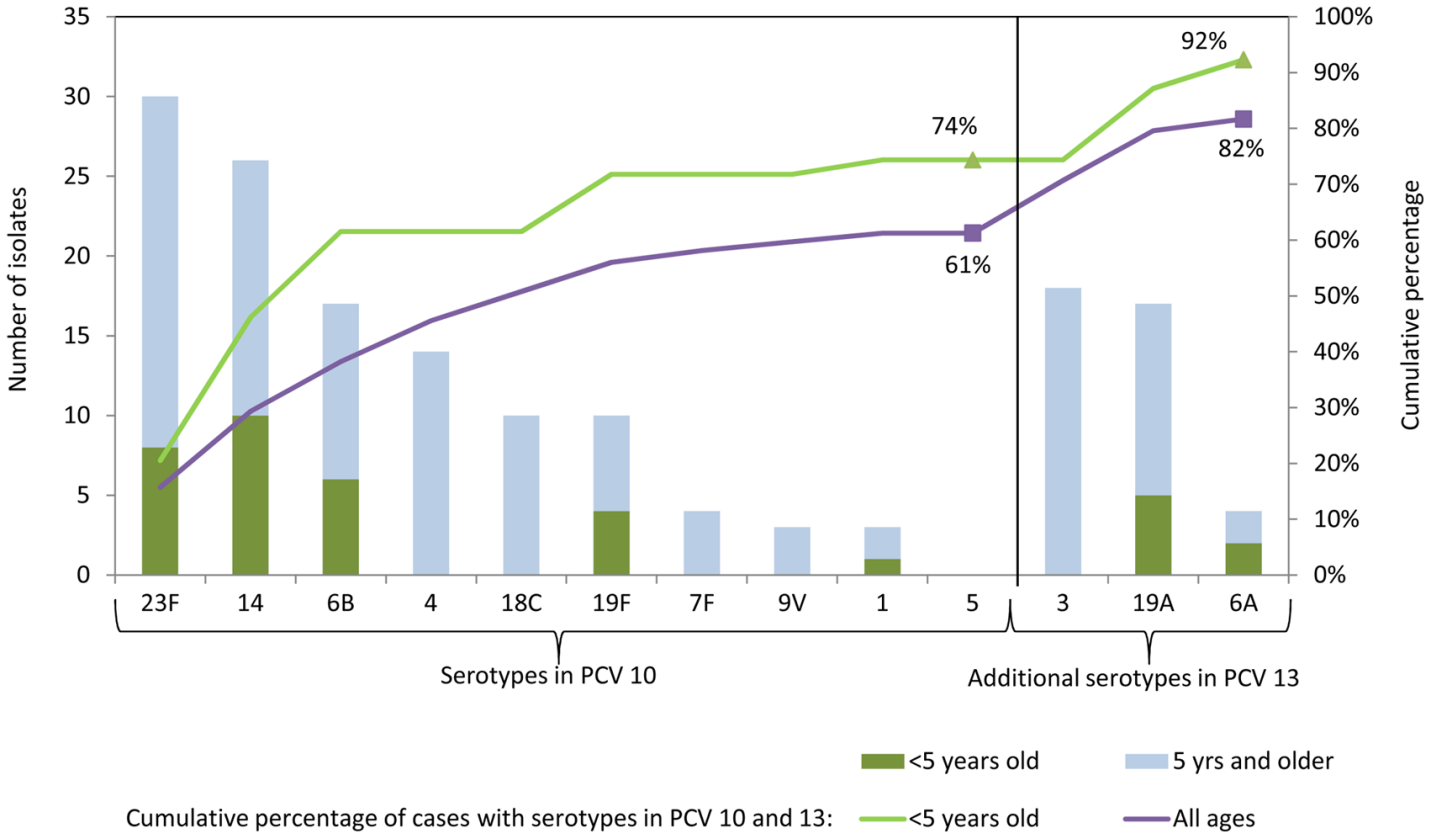
Serotype distribution of isolates from pneumococcal bacteremia cases: limited to serotypes contained in PCV10 and PCV13*. * Non-vaccine serotypes included: 11A (5 cases); 35B (3 cases); 7C, 10A, 23A, 34, (2 cases each), 8, 13, 15A, 15B, 16F, 18F, 20, 22F, 23B, 24F, 31, 35C, 35F and 38 (1 case each). The serotypes for five cases were not identified by the multiplex PCR protocol and these isolates were not available for serotyping by other methods.

Among children aged <5 years, serotypes contained in the current 10- and 13-valent pneumococcal conjugate vaccines (PCV10 and PCV13) comprised 74%, and 92% of cases, respectively, exceeding the proportions among cases overall (Figure 3).

Antibiotic susceptibility testing was available for 193/196 isolates with the results as follows: co-trimoxazole non-susceptibility was 57% (n = 109); erythromycin 30% (n = 57); clindamycin 21% (n = 40); chloramphenicol 12% (n = 23). All isolates were susceptible to penicillin and cefotaxime. Non-susceptibility to 3 or more of the above antibiotics was observed in 18% (35/193). Isolates with serotypes included in PCV10 were more likely to be non-susceptible to co-trimoxazole, erythromycin, and clindamycin compared to non-PCV10 serotype isolates: 63% vs. 45% for co-trimoxazole; 42% vs. 10% for erythromycin; and 30% vs. 5.5% for clindamycin.

## Discussion

Based on 196 *S. pneumoniae* isolates collected during 4.4 years of bloodstream infection surveillance in 2 rural provinces, we estimated the overall incidence of hospitalization for pneumococcal bacteremia in rural Thailand at 3.5 per 100,000 person-years. Rates were highest among children <5 years old (11.1 per 100,000 person-years) and adults 65 years and older (13.6 per 100,000 person-years).

These findings demonstrate that *S. pneumoniae* is an important cause of severe disease requiring hospitalization in Thailand. However, these data certainly underestimate the true incidence of pneumococcal bacteremia. First, we only captured hospitalized cases, and data from the United States suggest that most pneumococcal bacteremia cases in young children occur among outpatients [14]. Second, despite increased use of blood cultures since implementation of automated blood culture processing in 2005, many patients who would likely have blood culture performed in higher resource settings do not receive them in rural Thailand. From May 2005 through June 2007, only 66% of patients with indications for blood culture had a culture performed and for patients <5 years old the proportion was just 47% [6]. Furthermore, pre-culture antibiotic use remains common in this setting. We recently examined this issue and estimated that pre-culture antibiotics reduced our pneumococcal bacteremia incidence rates by 32% overall and 39% in children <5 years of age [12]. Finally, our surveillance does not include other manifestations of invasive pneumococcal disease, such as meningitis, arthritis or osteomyelitis.

These incidence estimates are comparable to our previously reported estimates examining 72 *S. pneumoniae* isolates from 23,853 blood cultures performed from May 2005 through June 2007 [6]. However, our previous report included estimates based on a combination of cases identified via *S. pneumoniae* isolation and cases identified only by Binax NOW® immunochromatographic test (ICT) on broth of blood cultures that had a positive signal in the BactT/ALERT® machine but were negative on sub-culture (alarm positive, sub-culture negative). The current report does not include these ICT-only cases, because more recent investigations indicate false-positive tests can occur [15] and we are formally evaluating this unlicensed application of ICT. Alarm positive, sub-culture negative bottles continue to pose a dilemma in our laboratories; from January through March 2010, 89 (2.2%) of 3891 blood cultures were alarm positive, sub-culture negative.

The proportion of fatal cases in Nakhon Phanom province (12%) was comparable to that reported in other publications from Thailand: 8.2% from Siripongpreeda et. al. (all invasive pneumococcal disease), 16% from Netsawang et. al (non-meningitis), 13.3% (non-meningitis) in Suwanpakdee *et al*.[16]–[18]. By comparison, the case fatality rate in Sa Kaeo province (37%) seemed unusually high. Unfortunately, data detailing clinical characteristics, treatment, and underlying conditions were not available to investigate this unusually high case fatality rate. However, our data do suggest that severity of illness differed between the 2 provinces, with substantially more patients in Sa Kaeo requiring oxygen and intubation.

We documented consistent, statistically significant seasonal increases in pneumococcal bacteremia from December through March, which substantiates the seasonal increase noted in other reports from Thailand [16], [17], [19], [20]. This seasonal pattern coincides, approximately, with Thailand’s cool season (November through February) and the seasonal increases in pneumococcal disease observed in the U.S. and other temperate regions during the winter months [21], [22]. Interestingly, the pneumococcal bacteremia peaks in Thailand occurred during opposite times of year as Thailand’s usual influenza season [23], [24], which differs from temperate climates where invasive pneumococcal disease and influenza peaks coincide [25]. This report includes data during the 2009 influenza pandemic, which first peaked in Thailand from July to September 2009, during which time pneumococcal bacteremia rates were low (Figure 2).

We observed that a high proportion of pneumococcal bacteremia cases among children aged <5 years were caused by serotypes covered by PCV10, and that with the addition of PCV13 serotypes, coverage increases from 74% to 92% for children <5 years old and from 61% to 82% overall. In a 2010 report, Thai researchers in the Bangkok area found that a similarly high proportion of IPD cases among children aged <5 years were caused by vaccine serotypes: 70% and 81% for PCV7 and 13 respectively [26]. The Thailand National Institute of Health reported even higher proportions of vaccine serotypes among children aged <5 years with invasive disease (80% for PCV10 and 92% for PCV13) [27]. Taken together these findings provide strong evidence that high coverage could be expected from PCV13 in Thailand.

All pneumococcal isolates were sensitive to penicillin, although we observed high rates of antibiotic non-susceptibility to a variety of other drugs, which is in agreement with many reports from Thailand [16], [18], [19], [28] and the region [29]. Our finding that antibiotic non-susceptibility is significantly higher among PCV serotypes corroborates other reports from Thailand and suggests that enactment of PCV implementation could help reduce antibiotic non-susceptibility, as was seen in the U.S. after vaccine introduction [30], [31].

These findings document the ongoing burden of hospitalized pneumococcal bacteremia, which represents a small fraction of the total pneumococcal disease burden. In previous work among adults, we found that blood culture alone underestimates the incidence of hospitalized pneumococcal pneumonia cases by at least 9-fold [32]. Taken together with recent reports from other pneumococcal researchers in Thailand, our findings highlight the potential impact of PCV in Thailand and underscore the need for cost effectiveness data to inform vaccine policy discussions and decision making.

## References

[1] O’Brien KL, Wolfson LJ, Watt JP, Henkle E, Deloria-Knoll M, et al (2009) Burden of disease caused by *Streptococcus pneumoniae* in children younger than 5 years: global estimates. Lancet 374: 893–902. 19748398 10.1016/S0140-6736(09)61204-6

[2] Bravo LC (2009) Overview of the disease burden of invasive pneumococcal disease in Asia. Vaccine 27: 7282–7291. 19393708 10.1016/j.vaccine.2009.04.046

[3] WHO (2007) Pneumococcal conjugate vaccine for childhood immunization–WHO position paper. Wkly Epidemiol Rec 82: 93–104. 17380597

[4] Wongsawat J, Chokephaibulkit K (2010) Implication of pneumococcal conjugate vaccines to public health: Thailand perspective. J Med Assoc Thai 93: S53–S59. 21294383

[5] GAVI, World Bank (2012) Creating markets to save lives. Factsheet: Advance Market Commitment. Available: http://www.gavialliance.org/library/gavi-documents/amc/Accessed 2013 May 13.

[6] Baggett HC, Peruski LF, Olsen SJ, Thamthitiwat S, Rhodes J, et al (2009) Incidence of pneumococcal bacteremia requiring hospitalization in rural Thailand. CID 48 (Suppl 2)S65–S74. 10.1086/596484 19191621

[7] National Economic and Social Development Board of Thailand. Population Projections of Thailand 2000–2030. Available: http://www.nesdb.go.th/temp_social/pop.zip. Accessed 2008 May 20.

[8] Olsen SJ, Thamthitiwat S, Chantra S, Chittaganpitch M, Fry AM, et al (2010) Incidence of respiratory pathogens in persons hospitalized with pneumonia in two provinces in Thailand. Epidemiol Infect Epidemiology and infection 138: 1811–22. 20353622 10.1017/S0950268810000646

[9] Perilla MJ, Ajello G, Bopp C, Elliott J, Facklam R, et al.. (2003) Manual for the laboratory identification and antimicrobial susceptibility testing of bacterial pathogens of public health importance in the developing world. Geneva: World Health Organization.

[10] Pai R, Gertz RE, Beall B (2006) Sequential multiplex PCR approach for determining capsular serotypes of *Streptococcus pneumoniae* isolates. J Clin Microbiol 44: 124–131. 16390959 10.1128/JCM.44.1.124-131.2006 PMC1351965

[11] Clinical and Laboratory Standards Institute (CLSI). Performance standards for antimicrobial susceptibility testing : 18th information supplement. Wayne, PA: CLSI, 2008.

[12] Rhodes J, Hyder JA, Peruski LF, Fisher C, Jorakate P, et al (2010) Antibiotic use in Thailand: Quantifying impact on blood culture yield and estimates of pneumococcal bacteremia incidence. Am J Trop Med Hyg 83: 301–306. 20682872 10.4269/ajtmh.2010.09-0584 PMC2911175

[13] Department of Provincial Administration, under Thailand Ministry of Interior. Population by individual age. Available: http://www.dopa.go.th/hpstat9/inhouse.htm Accessed 2011 February 28.

[14] Robinson KA, Baughman W, Rothrock G, Barrett NL, Pass M, et al (2001) Epidemiology of invasive *Streptococcus pneumoniae* infections in the United States, 1995–1998: Opportunities for prevention in the conjugate vaccine era. JAMA 285: 1729–1735. 11277827 10.1001/jama.285.13.1729

[15] Baggett HC, Rhodes J, Dejsirilert S, Salika P, Wansom T, et al (2011) Pneumococcal antigen testing of blood culture broth to enhance the detection of Streptococcus pneumoniae bacteremia. Eur J Clin Microbiol Infect Dis 31: 753–6. 21822563 10.1007/s10096-011-1370-3

[16] Siripongpreeda N, Hattasingh W, Amornvipas P, Eampokalap B, Sakoolgnam S, et al (2010) Frequency and clinical course of invasive pneumococcal disease caused by penicillin-resistant and penicillin-sensitive *Streptococcus pneumoniae* in Thai children. J Med Assoc Thai 93 Suppl 5S1–S5. 21298829

[17] Netsawang S, Punpanich W, Treeratweeraphong V, Chotpitayasunondh T (2010) Invasive pneumococcal infection in urban Thai children: A 10 year review. J Med Assoc Thai 93 Suppl 5S6–S12. 21298830

[18] Suwanpakdee D, Samakoses R, Sirinavin S, Kerdpanich A, Simasathien S, et al (2010) Invasive pneumococcal disease in Phramongkutklao Hospital 2004–2008: Clinical data, serotype distribution and antibicrobial resistance patterns J Med Assoc Thai. 93 Suppl 5S40–S45. 21294380

[19] Srifuengfung S, Chokephaibulkit K, Tribuddharat C, Comerungsee S (2010) A description of antimicrobial susceptibility of *Streptococcus pneumoniae* - Siriraj Hospital, Thailand: 2008. J Med Assoc Thai 93 Suppl 5S27–S34. 21294379

[20] Sirinavin S, Vorachit M, Thakkinstian A, Hongsanguensri S, Wittayawongsruji P (2003) Pediatric invasive pneumococcal disease in a teaching hospital in Bangkok. Int J Infect Dis 7: 183–189. 14563221 10.1016/s1201-9712(03)90050-6

[21] Dowell SF, Whitney CG, Wright C, Rose CE, Schuchat A (2003) Seasonal patterns of invasive pneumococcal disease. Emerging Infectious Diseases 9: 573–579. 12737741 10.3201/eid0905.020556 PMC2972762

[22] Walter ND, Taylor TH, Dowell SF, Mathis S, Moore MR (2009) Holiday Spikes in Pneumococcal Disease among Older Adults. N Engl J Med 361: 2584–2585. 20032333 10.1056/NEJMc0904844

[23] Simmerman JM, Uyeki TM (2008) The burden of influenza in East and South-East Asia: a review of the English language literature. Influenza and Other Respiratory Viruses 2: 81–92. 19453467 10.1111/j.1750-2659.2008.00045.x PMC4634698

[24] Simmerman JM, Chittaganpitch M, Levy J, Chantra S, Maloney S, et al (2009) Incidence, seasonality, and mortality associated with influenza pneumonia in Thailand: 2005–2008. PLoS ONE 4(11): e7776 doi:10.1371/journal.pone.0007776 19936224 10.1371/journal.pone.0007776 PMC2777392

[25] Walter ND, Taylor TH, Shay DK, Thompson WW, Brammer L, et al (2010) Influenza circulaion and the burden of invasive pneumococcal pneumonia during a non-pandemic period in the United States. CID 50: 175–183. 10.1086/649208 20014948

[26] Srifeungfung S, Tribuddharat C, Comerungsee S, Chatsuwan T, Treerauthanaweeraphong V, et al (2010) Serotype coverage of pneumococcal conjugate vaccine and drug susceptibility of *Streptococcus pneumoniae* isolated from invasive or non-invasive diseases in central Thailand, 2006–2009. Vaccine 28: 3440–3444. 20199759 10.1016/j.vaccine.2010.02.071

[27] Dejsirilert S, Sirinavin S, Sawanpanyalert P, Saengsuk L, Polwichai P, et al.. (2010) A nationwide study on serotypes of invasive strains of pneumococcus in Thailand, 1998–2008. 7th International Symposium on Pneumococci and Pneumococcal Diseases Tel Aviv, Israel.

[28] Dejsirilert S, Tienkrim S, Ubonyaem N, Sawanpanyalert P, Aswapokee N, et al (2009) National antimicrobial resistance surveillance among clinical isolates of *Streptococcus pneumoniae* in Thailand. J Med Assoc Thai 92 Suppl 4S19–S32. 21298844

[29] Song JH, Jung SI, Ko KS, Kim NY, Son JS, et al (2004) High prevalence of antimicrobial resistance among clinical *Streptococcus pneumoniae* isolates in Asia (an ANSORP study). Antimicrob Agents and Chemother 48: 2101–2107. 15155207 10.1128/AAC.48.6.2101-2107.2004 PMC415617

[30] Hennessy TW, Singleton RJ, Bulkow LR, Bruden DL, Hurlburt DA, et al (2005) Impact of heptavalent pneumococcal conjugate vaccine on invasive disease, antimicrobial resistance and colonization in Alaska Natives: progress towards elimination of a health disparity. Vaccine 23: 5464–5473. 16188350 10.1016/j.vaccine.2005.08.100

[31] Kyaw MH, Lynfield R, Schaffner W, Craig AS, Hadler J, et al (2006) Effect of introduction of the pneumococcal conjugate vaccine on drug-resistant *Streptococcus pneumoniae* . N Engl J Med 354: 1455–1463. 16598044 10.1056/NEJMoa051642

[32] Baggett HC, Thamthitiwat S, Prapasiri P, Naorat S, Rhodes J, et al.. (2010) Incidence of pneumococcal pneumonia among adults in Thailand: Value of non-culture assays to enhance case detection. International Symposium on Pneumococcus and Pneumococcal Diseases (ISPPD) Tel Aviv, Israel.

